# Influence of Environmental Factors, Cultural Practices, and Herbicide Application on Seed Germination and Emergence Ecology of *Ischaemum rugosum* Salisb

**DOI:** 10.1371/journal.pone.0137256

**Published:** 2015-09-14

**Authors:** Charlemagne Alexander A. Lim, Tahir Hussain Awan, Pompe C. Sta. Cruz, Bhagirath Singh Chauhan

**Affiliations:** 1 Weed Science, Crop and Environmental Sciences Division, International Rice Research Institute (IRRI), Los Baños, Philippines; 2 Crop Science Cluster, College of Agriculture, University of Philippines, Los Baños, Phillipines; 3 The Queensland Alliance for Agriculture and Food Innovation, The University of Queensland, Toowoomba, Queensland, Australia; University of Vigo, SPAIN

## Abstract

*Ischaemum rugosum* Salisb. (Saramolla grass) is a noxious weed of rice that is difficult to control by chemical or mechanical means once established. A study was conducted to determine the effect of light, temperature, salt, drought, flooding, rice residue mulch, burial depth, and pre-emergence herbicides on seed germination and emergence of *I*. *rugosum*. Germination was stimulated by light and inhibited under complete darkness. Optimum temperature for germination was 30/20°C (97.5% germination). Germination reduced from 31 to 3.5% when the osmotic potential of the growing medium decreased from -0.1 to -0.6 MPa and no germination occurred at -0.8 MPa. Germination was 18 and 0.5% at 50 and 100 mM NaCl concentrations, respectively, but was completely inhibited at 150 mM or higher. Residue application at 1–6 t ha^-1^ reduced weed emergence by 35–88% and shoot biomass by 55–95%. The efficacy of pre-emergence herbicides increased with increasing application rates and decreased with increasing rice residue mulching. The efficacy of herbicides was in the order of oxadiazon> pendimethalin> pretilachlor. At 6 t ha^-1^, all herbicides, regardless of rates, did not differ from the control treatment. *I*. *rugosum* seeds buried at 2 cm or deeper did not emerge; however, they emerged by 4.5 and 0.5% at 0.5 and 1 cm depths, respectively, compared to the 39% germination for soil surface seeding. Flooding at 4 DAS or earlier reduced seedling emergence and shoot biomass while flooding at 8 DAS reduced only seedling emergence. The depth and timing of flooding independently reduced root biomass. Flooding at 4 and 6 cm depths reduced the root biomass. Relative to flooding on the day of sowing, flooding at 8 DAS increased root biomass by 89%. Similarly, flooding on the day of sowing and at 2 DAS reduced the root–shoot biomass ratio. Under the no-flood treatment, increasing rates of pretilachlor from 0.075 to 0.3 kg ai ha^-1^ reduced weed emergence by 61–79%. At the flooding depth of 2–4 cm, pretilachlor reduced weed emergence and shoot and root biomass, but the differences across rates were non-significant. Information generated in this study will be helpful in developing integrated weed management strategies for managing this weed.

## Introduction

Since the 1980s, many Asian farmers have shifted from transplanting to direct-seeding methods of crop establishment [1]. In developing countries, migration of labor to non-farm jobs, labor shortage, high cost for labor prompted many farmers to adopt direct-seeding methods [2]. Direct seeding is favored due to reduced costs owing to less labor requirements, early crop maturity (7–10 d earlier) due to rapid and easy crop establishment, less water consumption, availability of short-duration varieties and cost-efficient selective herbicides [3], and favorability of the system for mechanization [4]. Despite these, however, the risk for yield loss caused by weeds is higher in direct-seeded rice due to the absence of standing water during crop emergence and the lack of seedling size advantage of rice over weeds [5]. Water management and weed control are used to overcome these constraints [3].

*Ischaemum rugosum* Salisb. (saramolla grass) is one of the major weeds found in rice fields in Asia, particularly in Sri Lanka, India, Madagascar, Thailand, Fiji, and Surinam, and is a serious pest in the rice fields of Brazil, Ghana, Peru, the Philippines, Cambodia, Guinea, Liberia, Malaysia (Sarawak), Senegal, and Trinidad [6]. *I*. *rugosum* is an annual grass weed that is native to tropical Asia which can grow up to more than a meter high [7]. Once established in the field, *I*. *rugosum* is difficult to control by chemical or mechanical means [8]. At its vegetative stage, this weed resembles the rice (*Oryza sativa* L.) plant, making it difficult to distinguish [9]. It is considered as one of the noxious weeds by the United States Department of Agriculture (USDA) [10].

*I*. *rugosum* is a highly competitive weed in rice production as it was found to cause a 48% yield reduction in an experiment in Malaysia [11] and a 33% reduction in another trial in Colombia [12]. Another study in India showed that more than 25% loss in yield occurred when *I*. *rugosum* grew with transplanted rice for the first 40 d [13]. In the Philippines, only 5 plants per m^2^ of *I*. *rugosum* in the wet season and 20 plants per m^2^ in the dry season were needed to significantly reduce the yield of transplanted rice in the Philippines [14]. These studies suggest that *I*. *rugosum* is a serious weed in rice production systems.

Seed germination is an important process in determining the competitiveness of a weed in an agro-ecosystem [5], considering that weed seeds respond to different environmental factors such as temperature, light, soil salinity, soil moisture, and tillage and residue mulching practices [4]. Information on seed germination is also essential for the improvement of weed management systems for a specific weed species [15]. Further, tillage operations disturb the soil in various ways, depending on the farm implements used, which can affect weed populations [16]. Moldboard plow, for example, can invert the soil and bury the weeds below their maximum depth of emergence [16]. Crop residues when used as mulch can also suppress the emergence of some weeds [17, 18].

In transplanted rice, flooding suppresses weed emergence, hence, it has long been used as an effective control measure against several weeds. In direct-seeded systems, the need to grow rice under aerobic conditions allows weeds to establish, thus, rendering early flooding ineffective [19]. The use of chemical pesticides has been one of the major contributors to agriculture for the past decades [20]. Herbicide use, in particular, is the most preferred weed management method, especially in large-scale rice farming [21].

Effective weed management systems rely heavily on detailed knowledge on weed seed biology [4, 22]. Knowledge on the response of *I*. *rugosum* to environmental factors and common practices employed in weed management will bring a broader perspective on how to holistically and effectively manage this weed. However, limited information is available on the impact of environmental, cultural, and herbicidal factors on the seed germination and emergence of this weed. Hence, this study was conducted to i) determine the effect of environmental factors such as light, temperature, salinity, and drought on *I*. *rugosum* seed germination; and ii) to evaluate the emergence and growth responses of *I*. *rugosum* seedlings to weed management practices such as seed burial depth, mulching, flooding, and the use of different herbicides.

## Materials and Methods

### Seed collection, time, and place of study

The study was conducted at the laboratory and screenhouse facilities of the International Rice Reseach Institute (IRRI), Los Baños, Laguna, Philippines from November 2012 to June 2013.

In 2011, mature plants of I. rugosum were collected from the UI field of IRRI. Seeds collected from these plants were planted and grown in a screenhouse. Matured seeds from at least 200 plants were collected and cleaned. Seeds were placed in a paper bag and stored at room temperature in a laboratory. These seeds were labeled as the SC population and used them in the experiments. Again, in August 2012, mature seeds of I. rugosum were collected from at least 500 plants grown in the UI field of IRRI. These seeds were labeled as the UI population. Seed contaminants were removed from the seedlot. To prevent bacterial and fungal contaminations, seeds were soaked in 5.25% sodium hypochlorite solution for 5 min and rinsed with running tap water for 3–5 min before any germination or emergence test was conducted.

### Preliminary seed germination and seedling emergence tests

Using the clean seeds, germination test was carried out by placing 25 intact seeds (with hull) in a 9-cm diameter Petri dish lined with two pieces of Whatman no. 1 filter paper (Whatman International Ltd., Maidstone, U.K.) moistened with 5 mL distilled water or treatment solution. The side of the Petri dishes was sealed with parafilm. The Petri dishes were placed inside an incubator with fluctuating day/night temperatures of 30/20°C (light/dark), with photoperiod set at 12 h to coincide with the day and night interval.

The experiment on seedling emergence was conducted under screenhouse conditions. Three small plastic trays (8 cm x 8 cm x 5.5 cm) with small holes at the bottom were filled with sterilized soil and 25 intact seeds were sown on top, and then covered with a thin layer (≤ 0.1 cm) of the sterilized soil. The small trays were placed inside a larger tray filled with a small amount of tap water to saturate the soil. The setup was placed inside the screenhouse with an overhead transparent plastic sheet cover to protect the trays from rainfall.

These tests were conducted twice at a 4-week interval until the seedlot reached 80% germination and emergence. Germination was considered to have occurred when there was a visible protrusion of the radicle. On the other hand, emergence was considered when there was a visible plumule emerging from the soil surface. Seeds that germinated in the Petri dishes and emerged in the plastic trays were counted starting at 3 d after sowing (DAS) until no further germination or emergence was observed.

For the SC population, seeds were at least 8 months old before they were tested for germination while the UI seeds were tested at 2 months after collection. In the preliminary germination test, a low level of dormancy was observed for the SC seeds as seed germination was almost 100%. On the other hand, the UI population exhibited low germination (68%), which did not improve.

### Effect of light and temperature on germination

To determine the effect of light regimes and varying temperature on germination, 25 intact seeds were placed inside a 9-cm diameter Petri dish lined with two pieces of filter paper moistened with 5 mL distilled water. The Petri dishes were incubated in growth chambers with fluctuating day/night temperatures (35/25, 30/20, and 25/15°C) and different light regimes (light/dark for day/night environment and dark for completely no light environment). For the dark regime, the Petri dishes were wrapped with three layers of aluminum foil to ensure that no light would penetrate. The experiment with four replication was conducted in December 3, 2013 and repeated in December 17, 2013.

### Effect of salt stress on germination

To simulate salinity, sodium chloride (NaCl, Mallinckrodt Baker Inc., Phillipsburg, NJ) amounting to 0, 0.146, 0.242, 0.584, 0.877, 1.169, and 1.461 g was dissolved in 0.1 L water to obtain final NaCl concentrations of 0, 25, 50, 100, 150, 200, and 250 mM, respectively. These solutions were used to moisten the 25 intact seeds sown in each Petri dish. The Petri dishes were placed inside an incubator with fluctuating day/night temperatures of 30/20°C (light/dark), with photoperiod set at 12 h to coincide with the day and night interval The experiment with four replication was conducted in March 20, 2013 and repeated in April 4, 2013

### Effect of simulated drought (osmotic stress) on germination

To simulate drought, 0, 4.82, 6.815, 9.635, 11.8, and 13.625 g of polyethylene glycol 8000 (Sigma Aldrich Co., St. Louis MO) were dissolved in 0.05 L distilled water to achieve the solutions with osmotic potentials of 0, -0.1, -0.2, -0.4, -0.6, and -0.8 MPa, respectively (Michael, 1983). These solutions were used to moisten the 25 intact seeds sown in each Petri dish. The Petri dishes were sealed with parafilm and placed inside growth chambers with fluctuating day/night temperatures of 30/20°C (light/dark). The fluctuating temperatures imposed in the salinity and drought experiments were the optimum day/night temperatures for germination based from the results of the light regime and temperature test. The experiment with four replication was conducted in March 20, 2013 and repeated in April 4, 2013

For all experiments, the germinated seeds were incubated at 30/20°C (optimum day/night temperature identified from light and temperature germination test) and counted at 3-day intervals up to 15 DAS. For the seeds sown under the light/dark regime, germination was counted at 3-day intervals while for the seeds sown under the dark regime, germination was observed only at 15 DAS. At 15 DAS, the seeds sown at 250 mM NaCl (highest salinity level) and at -0.8 MPa osmotic potential (highest concentration) were rinsed with running water for 5 min, resown in new Petri dishes lined with two filter papers moistened with 5 mL of distilled water. Germination count was observed at 15 DAS and was expressed as percent based on seed germination count using the formula:

Percent germination=[Number of seeds that germinated/Number of sown seeds]×100

### Effect of seed burial depth on emergence and biomass of seedlings

Experiments were conducted inside a screenhouse (a 10 m x 20 m chamber framed with a 2-mm iron mesh and covered with a transparent plastic sheet to avoid rain damage) and 15-cm diameter pots were used as experimental units. The soil used was collected from the upland rice fields of IRRI and was analyzed, sterilized in an autoclave, and sieved through a 3-mm sieve. To determine the effect of soil burial depth on *I*. *rugosum*, each pot was lined at the bottom with a piece of paper to hold the soil inside and 25 intact seeds were sown on the soil surface then covered with soil at depths of 0, 0.5, 1, 2, 4, and 6 cm. The soil was kept saturated by placing the pots inside a large tray (30 cm x 24 cm x 10 cm) filled with water just enough to saturate the soil by capillary action without flooding the weed seeds. The pots were placed on benches inside the screenhouse until the end of each experimental setup. The pot surface was moistened regularly using a mist sprayer. The experiment with four replication was conducted in November 16, 2012 and repeated in November 30, 2012.

### Effect of amount of rice residue on emergence and biomass of seedlings

To determine the effect of rice residue cover, 25 intact seeds were sown on the soil surface in each pot. Straw (leaves and stem) of the rice variety NSIC Rc222 was finely chopped and weighed to 0, 1.77, 3.53, 7.07, and 10.60 g residue per pot and then spread evenly on the soil surface to simulate 0, 1, 2, 4, and 6 t ha^-1^ of residue cover, respectively. Seedling emergence was observed at 3-day intervals until no further emergence was observed. On the last day of observation (21 DAS), aboveground biomass was measured after samples were oven-dried at 70°C for 72 h. The experiment having four replication was conducted in November 16, 2012 and repeated in November 30, 2012.

### Response of *I*. *rugosum* seedlings to the interaction of pre-emergence herbicides and rice mulch

Plastic pots (15 cm diameter x 15 cm height) were lined with a paper at the bottom and filled with the same batch of soil as in the other tests and 25 intact seeds were sown in the pots. Straw (leaves and stem) of the rice variety NSIC Rc222 was finely chopped and weighed to 0, 3.53, and 10.60 g residue per pot and spread evenly on the soil surface to simulate 0, 2, and 6 t ha^-1^ of residue cover, respectively. The pots with the residue were placed inside trays (58 cm x 36 cm x 24 cm) which were filled with water to saturate the soil. The rice residue on top of the pots was moistened with water using a mist sprayer on the day of sowing. Using a research track sprayer (DeVries Manufacturing, Hollandale, MN), the following pre-emergence herbicides were applied at 1 DAS: oxadiazon (Bayer Crop Science, Laguna, Philippines) at 500 and 1000 g ai ha^-1^; pendimethalin (BASF Philippines, Incorporated, 11/F HHIC Building, 1128 University Parkway North Bonifacio, 1634 Global City, Taguig, Metro Manila, Philippines) at 1000 and 2000 g ai ha^-1^; and pretilachlor + safener (Syngenta Inc., 8th Floor Saustiana Dee Ty Tower 104 Paseo de Roxas Avenue) at 300 and 600 g ai ha^-1^. The track sprayer had a spray volume delivery of 210 L ha^-1^ and a 140 kPa spray pressure, fitted with a flat nozzle (Teejet 80015). One treatment was not sprayed with any herbicide to serve as the control. After the herbicide application, the pots were transferred to benches inside the screenhouse. The surface of the pots were regularly moistened with water using a mist sprayer. Seedling emergence was observed at 7, 14, and 21 d after herbicide application. At 21 d after herbicide spray, roots and shoots were harvested, oven-dried at 70°C for 72 h, and weighed to obtain dry biomass weight. The experiment with four replication was conducted in January 8, 2013 and repeated in January 22, 2013

### Response of *I*. *rugosum* seedlings to flooding depth and flooding time

Small plastic trays (8 cm x 8 cm x 5.5 cm) were filled with the same batch of soil as in the burial depth and residue experiments. The trays have four small holes at the sides for water to enter while tightly holding the soil inside. Fifty intact seeds of *I*. *rugosum* were sown on the top of the small trays. The small trays were placed inside larger plastic trays (14.5 cm x 14 cm x 11.5 cm) that were marked on their sides with 0, 2, 4, and 6 cm from the soil surface level of the small trays to indicate flooding depth. The soil in the smaller trays was kept saturated until flooding (based on treatments started) which was given at 0, 2, 4, and 8 DAS and was maintained for 21 d. Seedling emergence was observed at 14 and 21 d after flooding. At 21 d after flooding, roots and shoots (leaf and stem) were harvested and oven-dried at 70°C for 72 h to measure dry biomass. The experiment with four replication was conducted in November 21, 2012 and repeated in May 7, 2013

### Seedling response of *I*. *rugosum* to different doses of pretilachlor and flood depth

Fifty intact seeds of *I*. *rugosum* were sown in pots (9 cm x 8 cm x 9 cm) containing the same soil as described in the previous experiments. The pots were saturated with water for 24 h on the day of sowing. At 1 DAS, pretilachlor + safener was sprayed at 0, 75, 150, 225, and 300 g ai ha^-1^ using the research track sprayer. The pots were transferred to the benches inside the screenhouse, were saturated after the spray, and surface-moistened with a mist sprayer. After 48 h of spray, the pots were placed in a larger leak-free circular canister (5.5 cm diameter and 12 cm high) and were flooded. The canisters were marked 0, 2, and 4 cm from the soil surface of the pots to indicate flooding depth. Seedling emergence was observed at 14 and 21 d after spray. At 21 d after spray, roots and shoots were harvested, oven-dried at 70°C for 72 h, and weighed to obtain dry biomass. The experiment having four replication was conducted in November 25, 2012 and repeated in May 14, 2013

### Experimental design and statistical analyses

For the experiments, each replication was arranged on different shelves in the incubator in the laboratory or on different benches in the screenhouse which were considered as a block. All of the experiments were laid out in a randomized complete block design (RCBD) with four replicates, and were conducted two times. All the experiments were conducted using RCBD. As for experiments with two factors, they were conducted still in RCBD but all of the treatments (treatment A, treatment B, and the combination of both treatments A and B (AxB) were completely randomized in a block (replication). We can say the design for experiments with two factors is RCBD.

Analysis of variance (ANOVA) was performed to evaluate the interaction between treatments and experimental runs. Treatment means was compared using the least significant difference (LSD) test at *p* ≤ 0.05 [STAR (Statistical Tool for Agicultural Research) version 1.0 2013].

No interaction between the treatments and experimental runs was observed, therefore, the data were pooled over the two runs for further analysis. Before statistical analysis, homogeneity of variance was visually confirmed by inspecting the residuals.

Germination (as affected by temperature, and water and salt stress), emergence (as affected by rice residue quantity, and flood timing and depth), and shoot and root biomass (under different flood durations and depths) were analyzed using regression analysis, and modeled using a three-parameter sigmoid function. The fitted model was:

Y=Ymax/{1+exp[−(x−T50)/Yrate]}

where, *Y* is the cumulative germination, emergence, or biomass at time/depth/quantity *x*, *y*_*max*_ is the maximum parameter, *T*_50_ is the x-x value for 50% of maximum parameter, and *y*_*rate*_ indicates the slope for parameter.

An exponential model with two parameters,

y=a*exp(−b.x)

was fitted to the germination (affected by water and salt stress at 15 DAS), seedling emergence (affected by pre-emergence herbicides and rice residue quantity, and flooding depth and pretilachlor rates), shoot biomass (affected by burial depth, residue quantity, flood depth, and flooding depth and pretilachlor rates), and root biomass (obtained at different flooding depths, and flooding depth and pretilachlor rates) data. Where, *Y* is the estimated parameter, *a* is the maximum of the parameter, and *b* is the slope. Parameter estimates for each model were compared using their standard errors.

A linear model

y=a+b.x

was fitted to the seedling emergence affected by burial depth, shoot and root biomass (g) affected by residue quantity, and root to shoot weight ratio affected by flood timing, where, *y* is the predicted emergence or biomass as a function of residue quantity or DAS (x), *a* is the *y* intercept, and *b* describes the slope of the regression curve.

## Results and Discussion

### Preliminary seed germination and seedling emergence tests

The *I*. *rugosum* seeds used in this study came from two population sources, one population labeled as SC from the screenhouse, and another other labeled as UI from the IRRI upland fields. Seeds of the population UI showed lower germination at 30/20°C day/night temperature, less than 50% on the average, while seeds from the SC population had germination up to 92% in the same day/night temperature. Thus, seeds from the SC population were selected for the germination experiments in the laboratory.

Seeds from both populations had low seedling emergence when tested under the screenhouse conditions. Percent emergence for the population SC was only 52%, while the percent emergence of seeds from the population UI was 68%. Hence, seeds from the UI population were used in the experiment on seedling emergence affected by residue and pre-emergence herbicide. However, after 2 months and with further emergence tests, seeds from the SC population improved to 88% seedling emergence, while percent seedling emergence of seeds from the UI population did not improve. Therefore, seeds from the population SC were used in other remaining screenhouse experiments.

### Effect of light and temperature on germination

**Fig 1:**
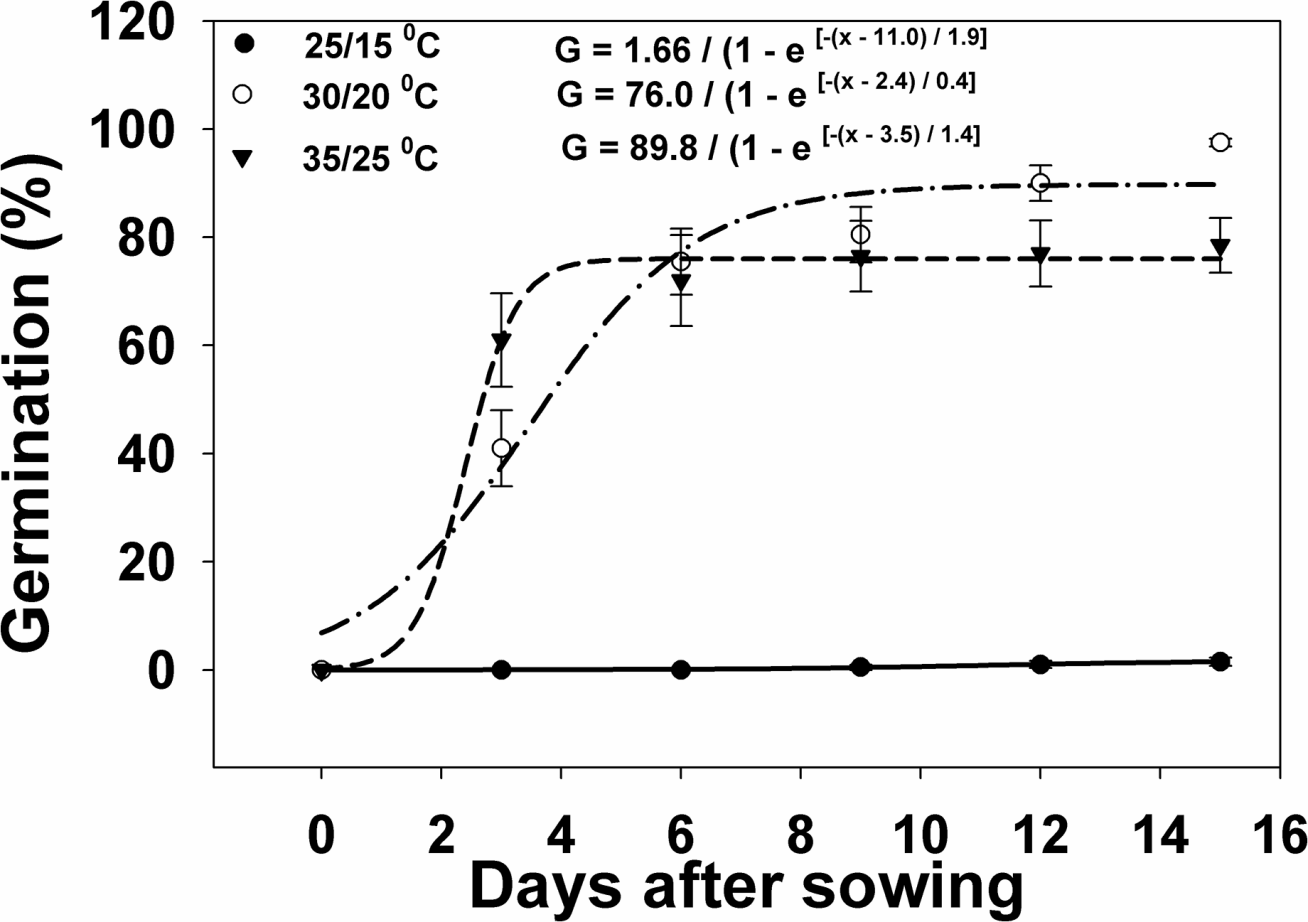
Germination response of *I*. *rugosum* as affected by temperature plotted over time (in days after sowing, DAS) modeled with the use of equation *G* = *G*_*max*_/(1 + e ^[- (*x - T50*)/*Grate*^ Vertical bars represent standard error of the mean.

Under light and dark regimes, increasing day/night temperatures greatly affected weed seed germination. At lower day/night temperatures (25/15°C), a maximum germination of 1.5% was observed at 15 DAS. At the 30/20°C temperature, seed germination increased from 41 to 97.5% within 3–15 DAS (Fig 1A). At the 35/25°C day/night temperature, germination was 61% within 3 DAS and increased to 78.5% at 15 DAS. The higher temperature in the 35/25°C regime may have accelerated seed germination early on but the overall seed germination was still relatively lower than the 97.5% germination at the 30/20°C regime. The highest day/night temperatures resulted in reduced seed germination, indicating that germination may have been adversely affected. Seeds kept in the dark at the 25/15°C temperature did not germinate. Only a few seeds (≤1%) kept in the dark germinated at the 30/20 and 35/25°C temperature regimes and only exhibited very short and tiny radicles without shoots or plumules (data not shown). Light, therefore, was needed for the *I*. *rugosum* seeds to germinate.

Cooler day/night temperatures did not promote the germination of *I*. *rugosum* seeds (Fig 1A). Higher fluctuating day/night temperatures (35/25°C), however, accelerated germination, although in low percentages. The optimum temperature was 30/20°C, thereby explaining the prevalence of this weed in the tropics. Although a small proportion of the seeds germinated under dark conditions, light was still needed to stimulate germination. Light induces photomorphogenesis when photoreceptors like phytochromes intercept light [23]. Light could be a limiting factor to *I*. *rugosum* germination when its seeds are situated in the deep layers of the soil or are totally covered with thick crop residue under field conditions.

Germination response to temperatures varies among weed species as some species can germinate under constant and/or fluctuating temperatures while others have enhanced germination with increasing fluctuations in temperature [24, 25]. The rate of emergence is highly correlated with soil temperatures, thus, temperature is critical in influencing the speed and occurrence of germination [17].

Germination in *Eleusine indica* was significantly higher in light/dark conditions (68–72%) than in dark conditions (17–25%) at 30/20 and 35/25°C [26]. Both light and temperature can influence seed dormancy and germination [23, 24, 25].

Germination dependence to light differs among species [27]. Far-red (FR) light inhibits seed germination through phytochromes by actively converting active forms of phytochromes (Pfr) into inactive forms (Pr) in phytochrome-controlled seeds. On the other hand, red (R) light promotes conversion of Pr to Pfr that results in germination [28, 29, 30]. In cropping systems, light can be manipulated to reduce the germination and emergence of weeds [31].For instance, the requirement of *Leptochloa chinensis* (L.) Nees for light in order to germinate is absolute, and the weed can be problematic in no-till cropping systems where weed seeds are concentrated on or are close to the soil surface [32].

### Effect of simulated drought (osmotic stress) on germination

**Fig 2:**
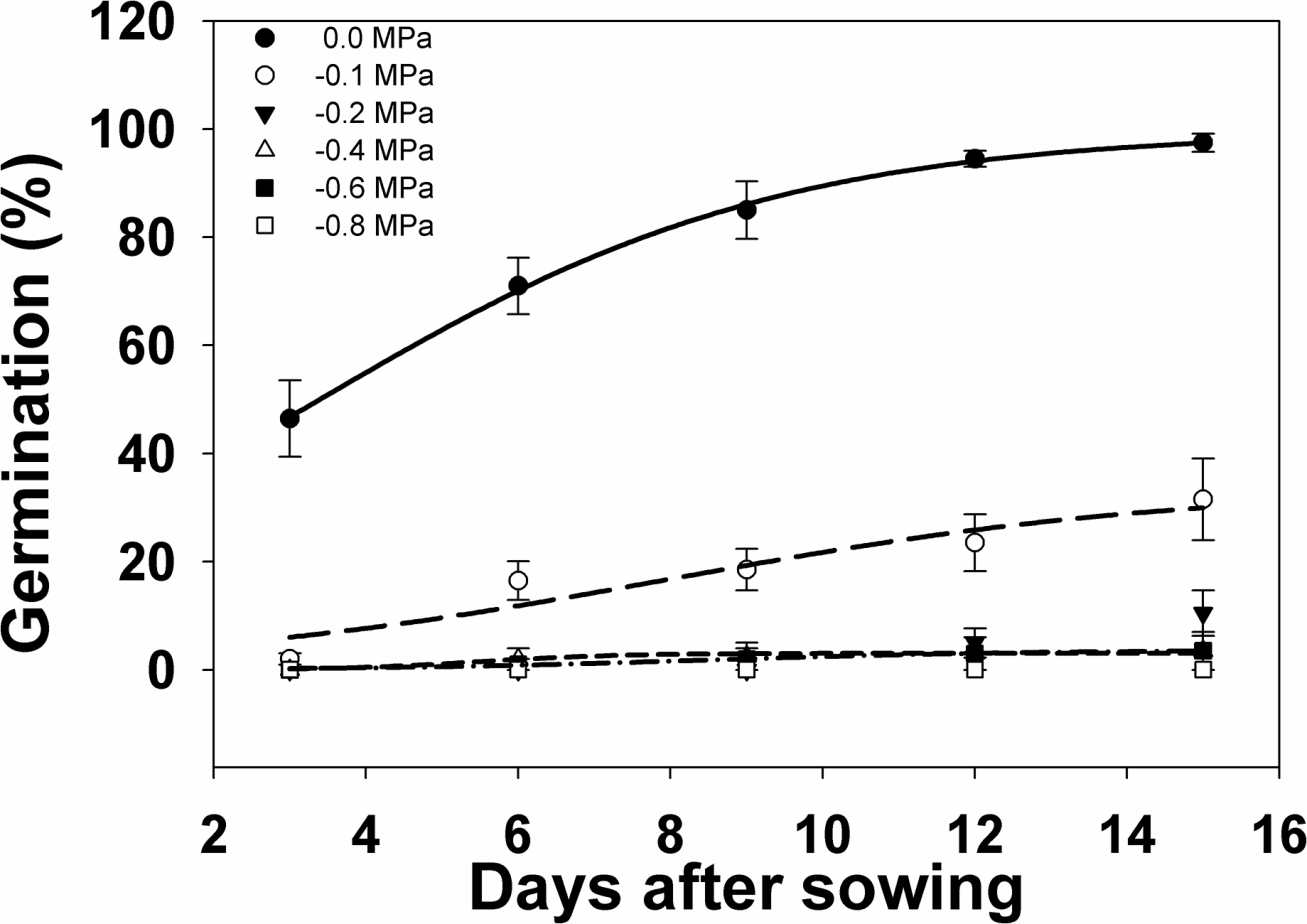
Germination percent response of *I*. *rugosum* as affected by decreasing osmotic potential (MPa) plotted over time (in days after sowing, DAS), modeled with the use of equation *G* = *G*_*max*_/(1 + e ^[- (*x - T50*)/*Grate*].^ Estimated parameters are given in Table 1. Vertical bars represent standard error of the mean; (b) Effect of different osmotic potential (MPa) on *I*. *rugosum* seed germination at 15 DAS modeled with the use of equation *G* = *G*_*max*_ x *e* ^(Grate . x)^. Vertical bars represent standard error of the mean.

Water stress greatly affected seed germination although a small proportion of seeds can still germinate at high water stress. At -0.1 MPa, only 31.5% of the seeds germinated at 15 DAS, which was significantly lower compared to the germination of 97.5% of the seeds sown in Petri dishes with no water stress at 15 DAS (Fig 2A, Table 1). Seeds sown on the Petri dishes with -0.4 and -0.6 MPa water stress had significantly very low germination at 3.5%. In the control treatment (0 MPa), seed germination reached 71% by 6 DAS, with more seeds germinating every 3-d interval.

**Table 1:** Effect of different osmotic potential (MPa) on germination of *I*. *rugosum* seed, incubated at 35/25°C in light/dark conditions. Parameter estimates [*G*_*max*_, maximum germination (%); *T*_*50*_, time to reach 50% of maximum germination (d); and *G*_*rate*_, slope] of a three-parameter sigmoid model {*G* = *G*_*max*_/(1 + e ^[- (*x - T50*)/*Grate*]^} fitted to seed germination data in Fig 1. Values in parentheses represent standard error of the mean.

Osmotic Potential of the medium (MPa)	Germination coefficients			*R* ^*2*^
	*G*_*max*_	*G*_*rate*_	*T*_*50*_	
0	99.53 (1.31)	3.03 (0.20)	3.36 (0.13)	0.99
-0.1	33.51 (12.04)	3.29 (2.32)	7.99 (3.27)	0.99
-0.2	Model could not fit			
-0.4	3.04 (0.30)	0.90 (0.79)	5.52 (0.70)	0.93
-0.6	3.61 (0.30)	2.09 (0.47)	8.48 (0.62)	0.99
-0.8	Model could not fit			

On the contrary, seed exposure to increasing levels of water stress resulted in a very low number of germinated seeds at 6 DAS, with fewer increases every 3-day interval. The seeds did not germinate at the -0.8 MPa water stress level. However, when these seeds were rinsed and resown in Petri dishes and moistened only with water, germination percentage reached 97.3% by 15 d after resowing (data not shown). This shows that despite subjecting the seeds to severe water stress beforehand, *I*. *rugosum* seeds can still germinate when provided with adequate moisture after exposure to severe water stress for 15 d. Thus, pre-exposure of *I*. *rugosum* seeds to severe water stress did not affect the ability of the intact seeds to germinate when a more favorable environment was present after water stress.

Our results conform with the earlier findings on *Urena lobata* wherein the osmotic potential required for 50% inhibition of the maximum germination was -0.1 MPa, although some seeds germinated at -0.8 MPa but none at -1.6 MPa [33]. A similar germination response was observed in *E*. *colona*, in which germination decreased from 80 to 1% as osmotic potential decreased from 0 to 20.8 MPa and was completely inhibited at osmotic potential of 21.0 MPa [34]. Seed germination decreased with reduced osmotic potential in *E*. *glabrescens*. Maximum seed germination (98%) was observed at 0 MPa. At osmotic potential of 20.2 MPa, seed germination *E*. *glabrescens* was 58–78%, respectively. An osmotic potential of 20.4 MPa reduced seed germination by 60–66%, respectively, compared with the control (i.e., 0 MPa). Some seeds germinated (4%) at 20.8 MPa, but germination was totally inhibited at 21.0 MPa. The osmotic potential required to inhibit 50% germination was 20.33 and 20.24 mM, respectively [35]

### Effect of salt stress on germination

**Fig 3:**
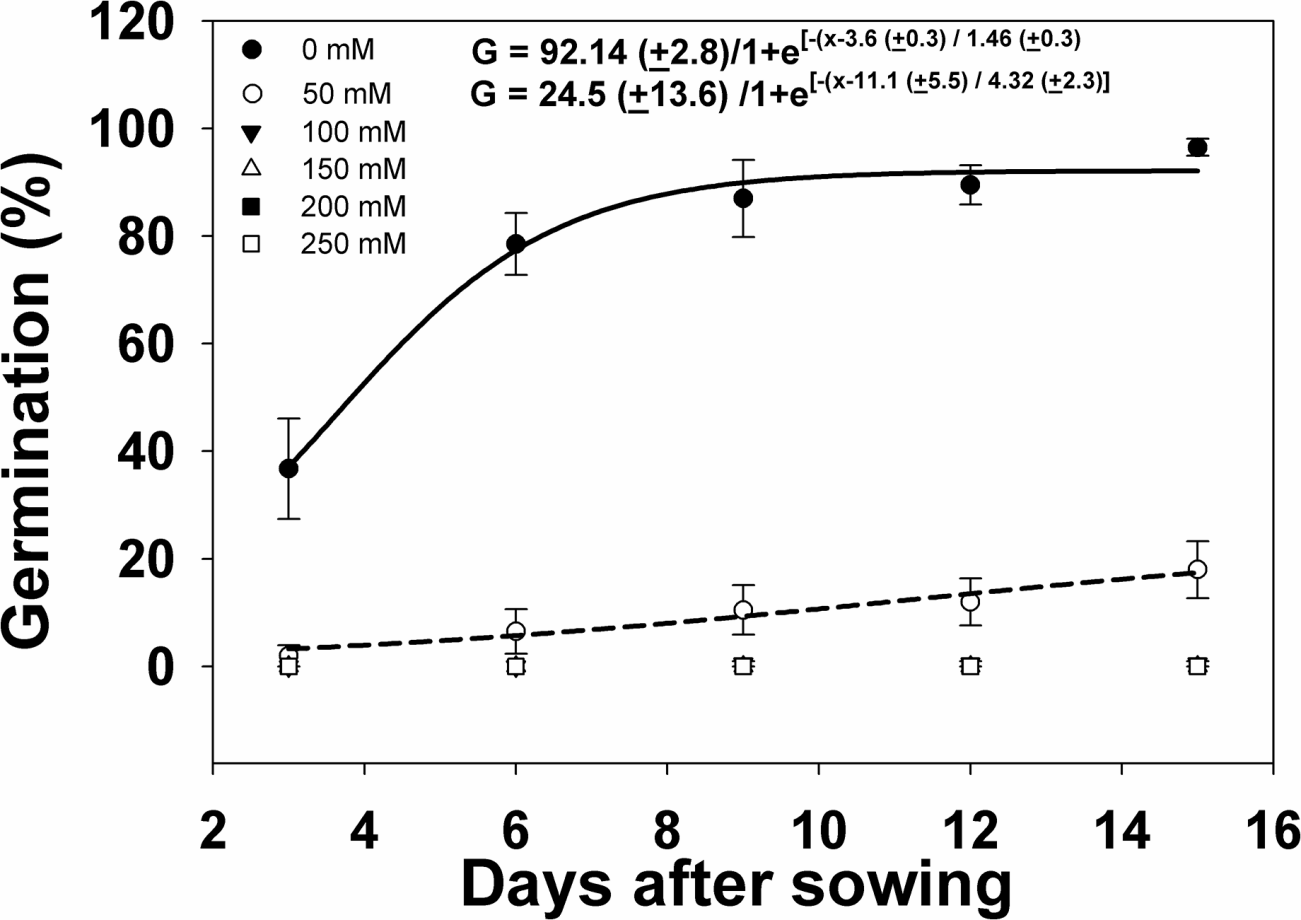
Germination percent response of *I*. *rugosum* as affected by NaCl concentration (mM) plotted over time (in d after sowing, DAS) modeled with the use of equation *G* = *G*_*max*_/(1 + e ^[- (*x - T50*)/*Grate*]^. Vertical bars represent standard error of the mean; (b) Effect of NaCl concentration (mM) on *I*. *rugosum* seed germination at 15 DAS modeled with the use of equation *G* = *G*_*max*_ x *e* ^(-Grate . x)^. Vertical bars represent standard error of the mean.

The increasing level of salt stress significantly reduced weed seed germination. When not subjected to salt stress, germination increased from 36.5 to 96.5% from 3 to 15 DAS (Fig 3A). Adding 50 mM of NaCl concentration reduced weed seed germination to only 2% at 3 DAS, which slowly increased to 18% by 15 DAS. At 100 mM NaCl concentration, only 0.5% of the seeds germinated at 9 DAS, with no further increase. Seeds subjected to salt concentrations of 150 mM or higher did not germinate.

The results showed that *I*. *rugosum* seeds are sensitive to salt stress even at a low concentration of 50 mM. However, when the seeds previously subjected to 250 mM of NaCl for 15 d were rinsed and resown in Petri dishes using water as a moistening agent, 82% germination was observed at 15 d after resowing. This indicates that, just like when the *I*. *rugosum* seeds were not subjected to water stress, they can readily germinate after severe salt stress when exposed to favorable environmental conditions.

However, this observation is not consistent among weed species that germinated under salt stress conditions. For example, *Ipomoea purpurea* L. had 40% reduction in germination when subjected to 50 mM NaCl, but still had 15% germination at 200 mM NaCl, indicating that *I*. *purpurea* can still persist in a field with up to 200 mM salinity level [31]. The range of salinity which *I*. *purpurea* can tolerate and the small proportion of seeds that could still germinate at high salinity levels can be important parameters that could enable the successful adaptation of this weed in saline areas [36]. Seeds of *Rapistrum rugosum* (L.) All. and *Sonchus oleraceus* L. had 11 and 7% germination, respectively, at 160 mM salt [37, 38]. Similarly, NaCl concentrations ranging from 0–200 mM affected the germination of *U*. *lobata* seeds significantly [33].

### Effect of seed burial depth on emergence and biomass of seedlings

**Fig 4:**
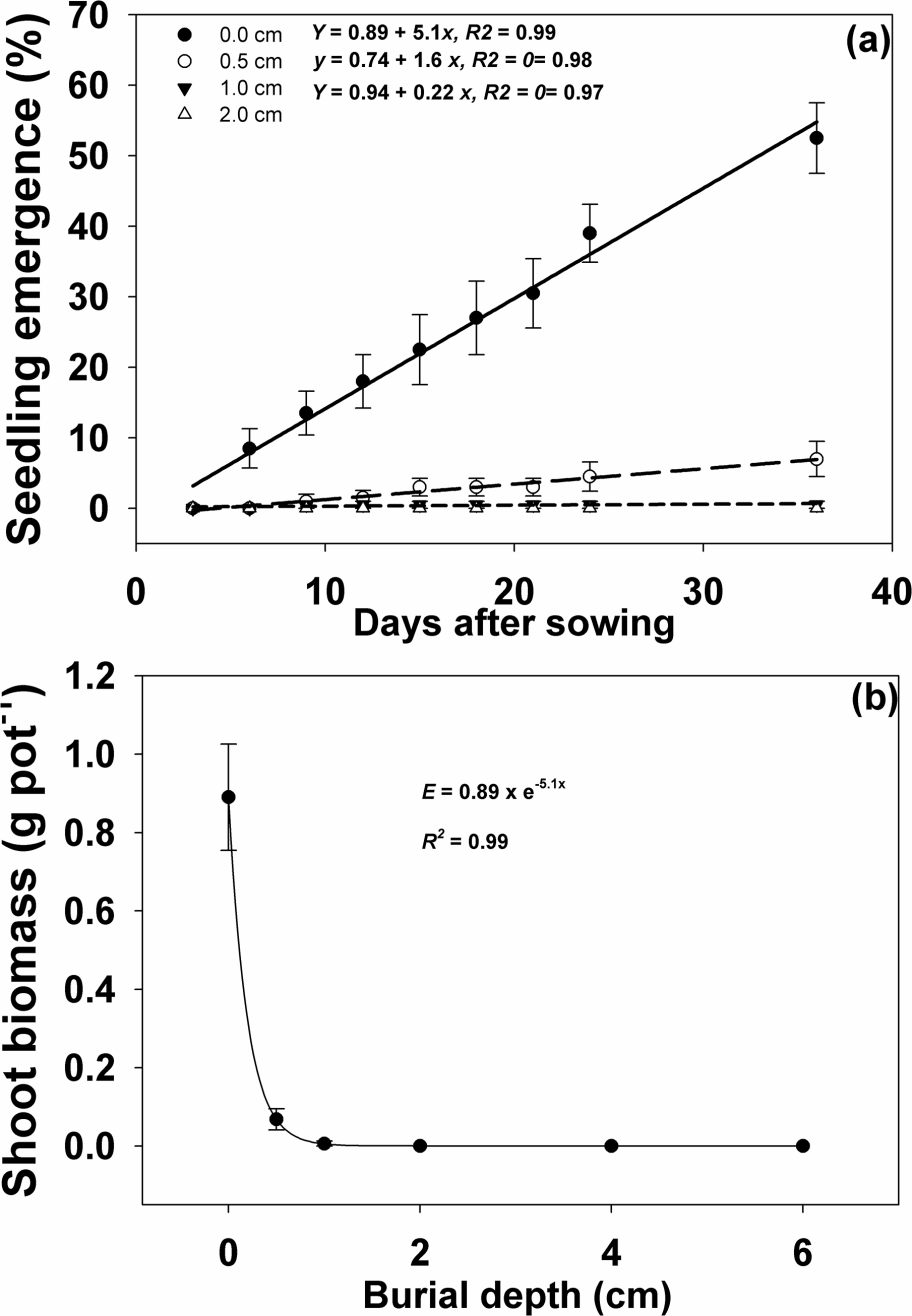
(a) Percent *I*. *rugosum* seedling emergence at varying burial depths (cm) observed at 3-day intervals until 24 d after sowing (DAS), modeled with the use of equation *y = a+bx*; (b) Effect of varying burial depths (cm) on *I*. *rugosum* shoot biomass (g pot^-1^) at 24 DAS modeled with the use of equation *G* = *G*_*max*_ x e (-*Grate* . *x*). Vertical bars represent standard error of the mean.

Deeper weed seed burial reduced the seedling emergence and shoot biomass of *I*. *rugosum* when compared to the control (surface seeding). Seed burial at a depth of 2 cm or deeper completely inhibited weed seedling emergence (Fig 4A). At 6 DAS, no emergence was observed for seeds buried at 0.5 cm or deeper. Weed emergence was significantly lower in seeds buried at 0.5 cm or deeper compared to the seeds sown at the soil surface from 9 to 24 DAS. At 24 DAS, weed seedling emergence was lower by 88 and 98% for seeds buried at 0.5 and 1.0 cm respectively, compared to the seeds sown on soil surface. Shoot biomass was also reduced by 91 and 99% for seeds buried at 0.5 and 1.0 cm deep, respectively, compared to the surface-sown seeds (Fig 4B). Clearly, burying *I*. *rugosum* seeds to a deeper soil layer reduces weed emergence and shoot biomass accumulation over time. Our results conform with an earlier study on the effect of the amount of rice residue on the germination of *U*. *lobata* seeds in which the burial depth required for 50% inhibition of maximum emergence was 2 cm and emergence was greatly reduced (93%) at burial depth of 4 cm or more. In a previous study [33], weed seedling biomass was found to decrease with increasing burial depth.

Tillage determines the distribution and burial of seeds along and across the soil profile [22]. In no-till farms, burial of seeds may depend on sowing equipment, wheel and animal traffic, soil cracks, and self-burial through structural seed features like hygroscopic awns [39]. Burial of seeds through tillage affects the weed population. For instance, densities of *Commelina benghalensis* L. were lower in conventionally-tilled fields than in reduced or minimally-tilled ones [40]. A decrease in seedling emergence with deeper burial depths was also observed in a previous study [4] and some species emerge at shallower burial depths, while emergence is drastically inhibited as the seeds are buried deeper. *Echinochloa colona* (L.) Link for instance, had 97% seedling emergence when sown at the surface, and 73, 12, and 0% emergence at 0.2, 0.5, and 6 cm burial depths, respectively [41]. Such a response was attributed to the photoblastic behavior of *E*. *colona* seeds in relation to the quality of light received under such depths. Less than 1% of incident light penetrates the soil at depths more than 0.2 cm [42, 43]. However, seedlings of some species like *Avena fatua* L., still emerge even from a depth of 20 cm [44]. Large seeds of *C*. *benghalensis* emerged from a depth of 5 cm but not from 10 cm, and small aerial seeds of the same plant failed to emerge beyond 1 cm burial depth [45].

Light and seed size are the usual limitations to seedling emergence from deep beneath the soil [5]. Some other factors that may also play a role include hypoxia due to low O_2_ and high CO_2_ concentrations as consequences of biological activity at deeper depths, decreasing germination and eventually emergence [46, 5, 47]; physical soil properties responsible for gas exchange with the external atmosphere [46]; and decreasing temperature fluctuations in deeper parts of the soil [48]. In the deeper layer of soil (deeper than 2cm) all above mentione factors contribute to the lower emergence of *I*. *rugosum*, *henece* Seed burial at a depth of 2 cm or deeper completely inhibited weed seedling emergence (Fig 4A).

### Effect of amount of rice residue on emergence and biomass of seedlings

**Fig 5:**
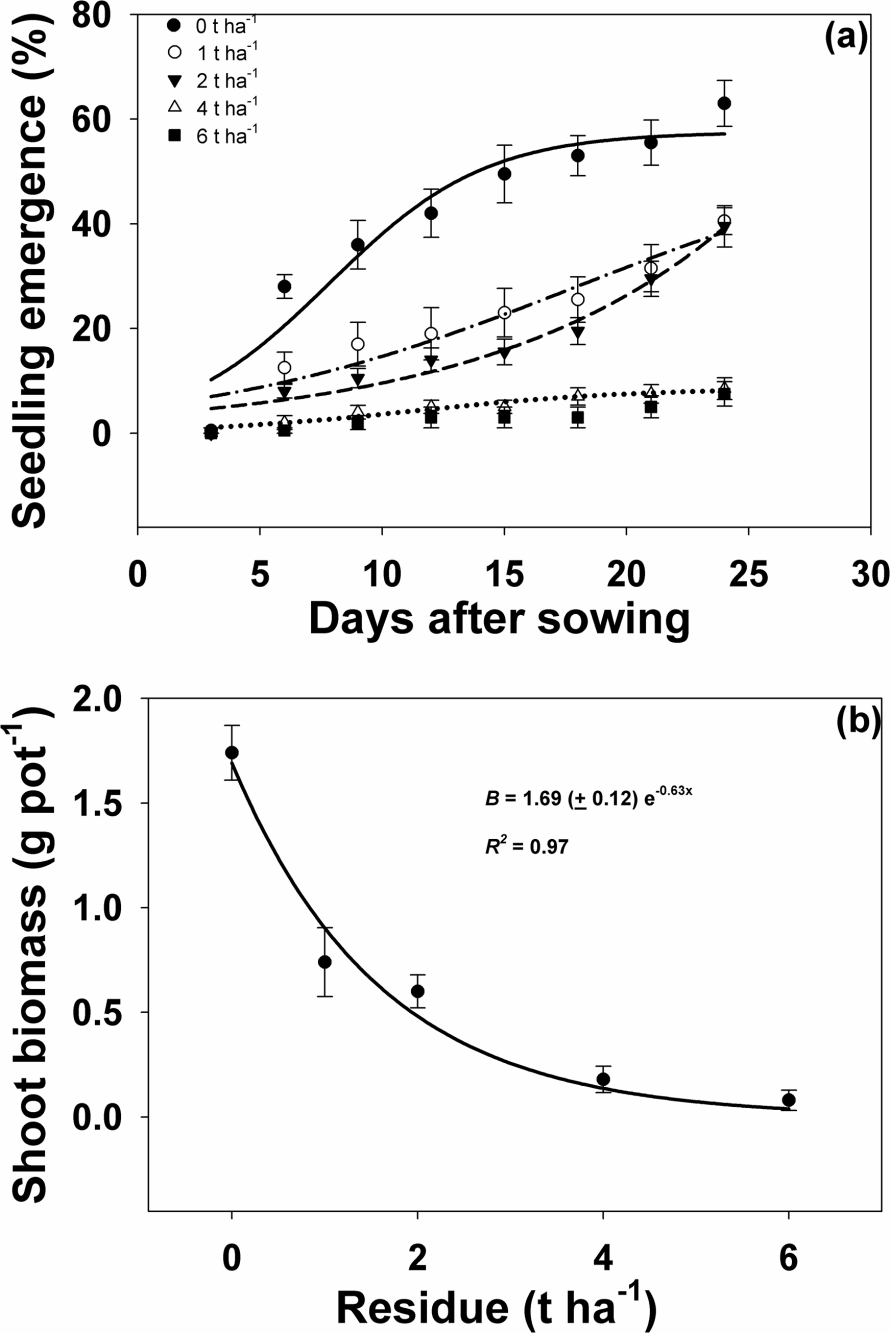
(a) Percent *I*. *rugosum* seedling emergence at different rice residue mulch observed at 3-day intervals until 24 DAS, modeled with the use of equation *G* = *G*_*max*_/(1 + e ^[- (*x - T50*)/*Grate*]^. Estimated parameters are given in Table 3. Vertical bars represent standard error of the mean; (b) Effect of amount of rice residue mulch on *I*. *rugosum* shoot biomass (g pot^-1^) at 24 DAS modeled with the use of equation *E* = *E*_*max*_ * *e* ^(-Erate . x)^. Vertical bars represent standard error of means.

Increasing the amount of rice residue used as mulch reduced *I*. *rugosum* seedling emergence and shoot biomass. Starting at 6 DAS, weed seedling emergence in pots with residue mulches were significantly lower compared to the seedling emergence in pots without residue mulch. This differential response continued to widen over time (Fig 5A, Table 2). However, the effect of 1 and 2 t ha^-1^ of residue mulch on weed emergence and shoot biomass was the same although significant compared to the control. The application of 4 and 6 t ha^-1^ of residue had a similar effect. At 24 DAS, 1 and 2 t ha^-1^ residue mulch application reduced weed seedling emergence by 35 and 37%, respectively, while 4 and 6 t ha^-1^ of rice residue mulch suppressed seedling emergence by 86 and 88%, respectively. In the same manner, weed shoot biomass was greatly reduced with increasing amounts of rice residue mulch (Fig 5B, Table 2), by 55 and 63% with 1 and 2 t ha^-1^ mulch applied, respectively, and by 89 and 95% with 4 and 6 t ha^-1^of residue mulching, respectively.

**Table 2:** Effect of rice residue amount (t ha^-1^) on seedling emergence of *I*. *rugosum* at various d after sowing. Parameter estimates [*E*_*max*_, maximum germination (%); *T*_*50*_, time to reach 50% of maximum emergence (d); and *E*_*rate*_, slope] of a three-parameter sigmoid model {*E* = *E*_*max*_/(1 + e ^[- (*x - T50*)/*Erate*]^} fitted to seedling emergence data in Fig 6. Values in parentheses represent standard error of the mean.

Straw residue quantity (t ha^-1^)	Seedling emergence coefficients			*R* ^*2*^
	*E*_*max*_	*E*_*rate*_	*T*_*50*_	
0	57.50 (4.59)	3.17 (1.08)	7.87 (0.0)	0.92
1	55.93 (32.48)	7.69 (3.80)	17.95 (9.84)	0.92
2	50 (30.08)	9.81 (4.17)	62.30 (386.33)	0.97
4	8.57 (1.13)	4.44 (1.39)	11.43 (1.80)	0.95
6	No fitting the curve			

The previous discussion showed that seedling emergence of *I*. *rugosum* was adversely affected by both burial depth and rice residue mulch (Figs 4 and 5). *S*eedling emergence was suppressed even at a burial depth of only 0.5 cm. The small seededness of *I*. *rugosum* seeds may explain the zero emergence observed at a depth of ≥ 2 cm. Seed size of this weed is relatively small as it accounts only about 20% of the whole seed structure together with its hull. Small seeds have lower ability to emerge from deeper burial depths mainly due to less energy reserves that will enable them to sustain the longer time to emerge [24]. Another possible reason for the lack of emergence may be that gases needed for germination could be limiting at deeper depths [43], thereby, also limiting seed germination.

Light could also account for the very low seedling emergence of *I*. *rugosum* in relation to burial depth and amount of residue. In the absence of light, seed germination may not take place. The observations on the seed burial depth and residue mulch experiment seem to agree with the observations in the light and temperature germination experiment where it was shown that light is required for the germination of *I*. *rugosum* seeds. Light spectrum that reaches the soil surface is highly affected by the presence of residues and canopies. Although light penetrates only a few mm in the soil, it can still be advantageous to small seeds at lower soil depths [42]. Dormancy may be induced in light-requiring seeds even in shallow depths by forcing absolute light requirement [49].

On the other hand, rice residue mulch can effectively control emergence and shoot biomass production of *I*. *rugosum* as it acts as a physical barrier to seedling emergence [41]. Hence, low residue levels in the field may not be enough to control weeds and high residue quantities may also not favor crop establishment. Under field conditions, a residue mulch of more than 2 t ha^-1^ is difficult to achieve and farmers may need to source out from other farms which would entail additional expenses on time, labor, and even transport. Such situation could limit farmers’ adoption of the use of rice residues as mulches. Moreover, the results under screenhouse conditions might differ considerably compared to results in actual field conditions, thus, studies to determine the effect of residue levels in the field remain crucial [41].

Crop residues when used as mulch can conserve soil and moisture in the soil and can suppress seedling emergence and growth of many weed species. Seedling emergence of *Amaranthus spinosus* L., *Echinochloa crus-galli* (P.) Beauv., *Dactyloctenium aegyptium* (L.) Willd., *Eclipta prostrata* (L.) L., *Digitaria ciliaris* (Retz.) Koel., *Eleusine indica* (L.) Gaertn., *Echinochloa colona*, and *Rottboellia cochinchinensis* (Lour.) W.D. Clayton is reduced as the amount of rice residue mulch is increased from 0 to 6 t ha^-1^ [4, 18]. In a field study, biomass production of *Eleusine indica* is least at 6 t ha^-1^ of rice residue, which is 89–95% lower compared to the treatment with no residue mulch [18]. The combined effect of weed species, allelopathic property of residue, and quantity and position of residue relative to weed seeds alter the immediate environment surrounding the weed seeds, resulting to the varying degrees of suppression that residue mulch effects on weed germination and growth [50].

### Response of *I*. *rugosum* seedlings to the interaction of different doses of pre-emergence herbicides and rice mulch

**Fig 6:**
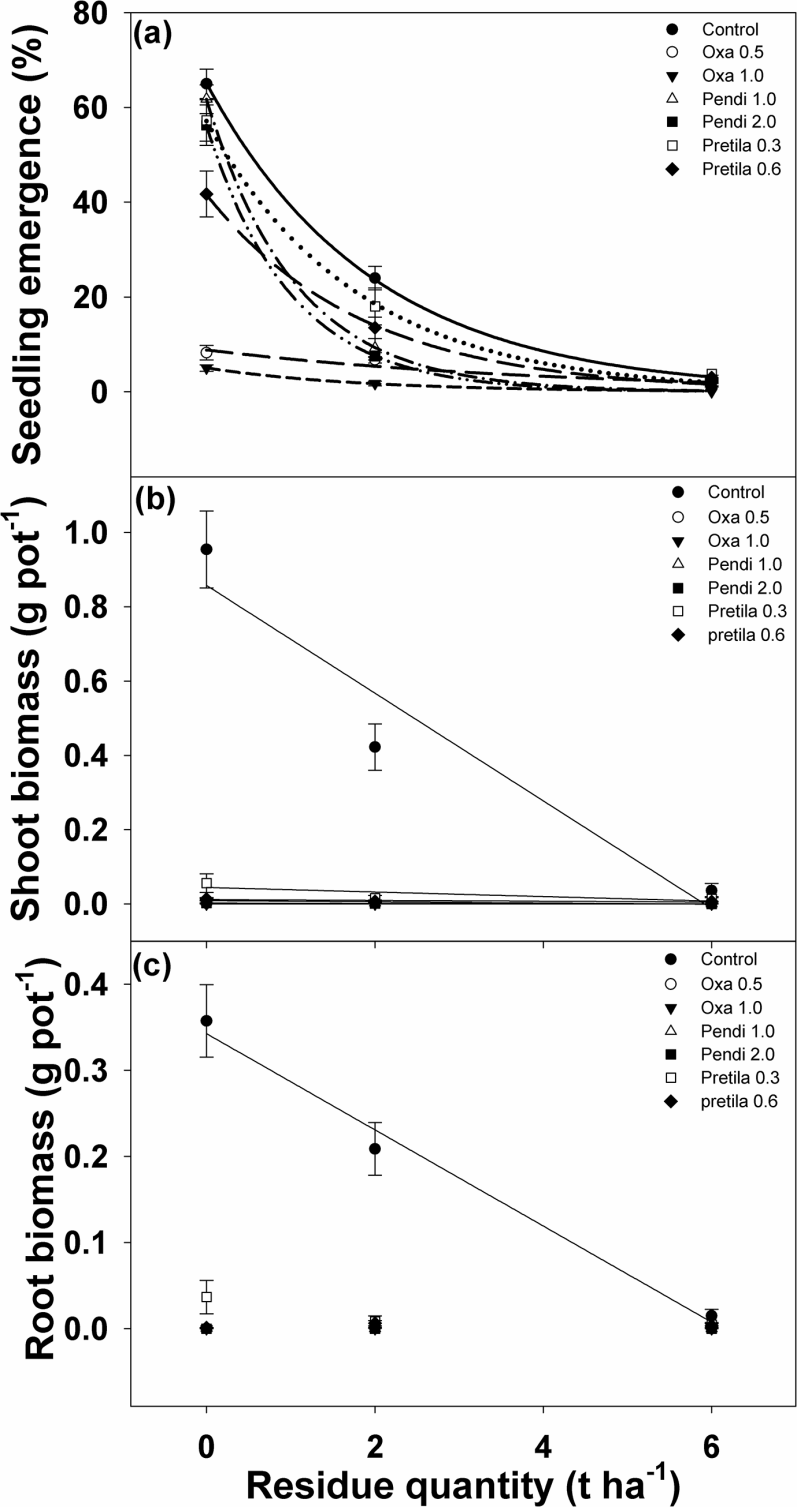
Interaction effect of pre-emergence herbicide and quantity of residue mulch (t ha^-1^) on (a) *I*. *rugosum* seedling emergence (%) modeled with the use of equation *G* = *G*_*max*_ x e (-*Grate* . *x*); (b) *I*. *rugosum* shoot biomass (g pot^-1^) and (c) root biomass (g pot^-1^) modeled with the use of equation *y = a + bx*. Estimated parameters are given in Table 2. Vertical bars represent standard error of the mean.

The effects of pre-emergence herbicides, amount of residue mulch, and their interactions on seedling emergence were significant (Fig 6, Table 3). Pre-emergence herbicide rates in combination with increasing amounts of residue mulch greatly reduced weed emergence. Under no residue cover, oxadiazon applied at 0.5 and 1.0 kg ai ha^-1^ reduced weed emergence by 87 and 92%, respectively, while pretilachlor applied at 0.6 kg ai ha^-1^ reduced it by 35%. Weed emergence in the pots treated with 1.0 kg ai ha^-1^ pendimethalin and 0.3 kg ai ha^-1^ pretilachlor did not differ with the control in the absence of residue mulch. At 2 and 6 t ha^-1^ of residue cover, however, 63 and 96% reduction on seed emergence, respectively, was observed compared to the no herbicide treatment. This shows the suppressing effect of pre-emergence herbicides and residue mulch on *I*. *rugosum* emergence, which is consistent with the results of the residue mulch experiment. Likewise, under 2 t ha^-1^ residue mulch, emergence in the pots treated with oxadiazon at 0.5 and 1.0 kg ai ha^-1^ and pendimethalin at 1.0 and 2.0 kg ai ha^-1^ differed significantly compared with the control, further reducing weed emergence by 65–92% and 61–68%, respectively. On the other hand, pretilachlor applied at 0.6 kg ai ha^-1^ reduced emergence by 43%, while pretilachlor at 0.3 kg ai ha^-1^ did not reduce weed emergence compared to the control with 2 t ha^-1^ residue cover. At 6 t ha^-1^ of residue cover, the effect of varying pre-emergence herbicide rates did not differ with the control.

**Table 3:** Effect of different preemergence herbicide rates (kg a.i. ha^-1^) on the seedling emergence of *I*. *rugosum* seeds. Parameter estimates [E_*max*_, maximum emergence (%) and E_*rate*_, slope] of a two-parameter exponential model [*E* = *E*_*max*_ * e ^(-Erate . x)^] fitted to seedling emergence data in Fig 4A. Parameter estimates [*y = a+bx*] of a Polynomial, Linear model fitted to shoot and root biomass data in Fig 4B and 4C. Values in parentheses represent standard error of the mean.

**Herbicide**	**Rate (kg a.i ha** ^**-1**^ **)**	**Seedling emergence coefficients**		*R* ^*2*^
		***E***_***max***_	***E***_***rate***_	
Control		65.08 (0.94)	0.51 (0.02)	0.99
Oxadiazon	0.5	8.81 (1.86)	0.25 (0.14)	0.88
Oxadiazon	1.0	5.01 (0.20)	0.55 (0.06)	0.99
Pendimethalin	1.0	61.74 (1.54)	0.94 (0.08)	0.99
Pendimethalin	2.0	56.24 (1.36)	1.00 (0.09)	0.99
Pretilachlor	0.3	57.13 (1.88)	0.56 (0.05)	0.99
Pretilachlor	0.6	41.64 (1.50)	0.55 (0.05)	0.99
		**Shoot biomass coefficients**		***R*** ^***2***^
		***a***	***b***	
Control		0.86 (0.15)	-0.15 (0.04)	0.93
Oxadiazon	0.5	0.0023 (0.00)	-0.0003 (0.00)	0.41
Oxadiazon	1.0	Model did not fit		
Pendimethalin	1.0	0.01 (0.00)	-0.002 (0.00)	0.75
Pendimethalin	2.0	0.0024 (0.00)	-0.0004 (0.00)	0.86
Pretilachlor	0.3	0.04 (0.02)	-0.01 (0.00)	0.61
Pretilachlor	0.6	0.01 (0.002)	-0.001 (0.00)	0.72
		**Root biomass coefficients**		***R*** ^***2***^
		***a***	***b***	
Control		0.34 (0.02)	-0.06 (0.01)	0.99
Oxadiazon	0.5	Model did not fit		
Oxadiazon	1.0	Model did not fit		
Pendimethalin	1.0	Model did not fit		
Pendimethalin	2.0	Model did not fit		
Pretilachlor	0.6	Model did not fit		

The application of pre-emergence herbicides combined with residue mulch significantly reduced the weed root and shoot biomass. Under no herbicide application, increasing the residue mulch from 2 to 6 t ha^-1^ reduced the root biomass by 39 and 95%, respectively (Fig 6C, Table 3). At 2 t ha^-1^ residue, both oxadiazon at 0.5 kg ai ha^-1^ and pendimethalin at 1.0 kg ai ha^-1^ reduced root biomass by 99%, and doubling their rates totally inhibited root biomass production, compared to the treatment seeds sown on bare soil. Pretilachlor at 0.3 and 0.6 kg ha^-1^ reduced biomass production by 83 and 99%, respectively, under bare soil and 96% for both rates at 2 t ha^-1^ residue mulch compared to the control. At 6 t ha^-1^ residue mulch, the effect of pre-emergence herbicides resulted in similar reductions on the weed root biomass relative to the control.

Without herbicide application, *I*. *rugosum* shoot biomass was reduced by 55 and 99% with 2 and 6 t ha^-1^ residue mulch, respectively (Fig 6B, Table 3). Under bare soil conditions, oxadiazon (0.5 and 1.0 kg ai ha^-1^) reduced shoot biomass by 99%, while pendimethalin applied at 1.0 and 2.0 kg ai ha^-1^ also significantly reduced shoot biomass by 98 and 99%, respectively. At 2 t ha^-1^ residue mulch, oxadiazon and pendimethalin significantly reduced shoot biomass by 99% compared to the control, while pretilachlor at 0.3 and 0.6 kg ai ha^-1^ reduced shoot biomass by 96 and 98%, respectively. At 6 t ha^-1^ residue mulch, however, all pre-emergence herbicides had similar reductions on weed shoot biomass as with the control.

Weed emergence was greatly affected by residue mulch and pre-emergence herbicide treatments. Although all the pre-emergence herbicides had effectively reduced the overall weed biomass, pendimethalin (both rates) and pretilachlor (0.3 kg ai ha^-1^) cannot effectively suppress weed emergence without residue mulch application. However, emergence under pendimethalin and pretilachlor treatments produced small seedlings with small and stunted plumule (3–5 mm) that did not completely form into a leaf or develop into a normal seedling. Based on this study’s criteria for emergence, these seedlings are considered to have emerged since the plumule is evident. The application of pre-emergence herbicides in combination with 2 t ha^-1^ residue mulch appeared to be more effective in reducing weed emergence compared to the application of rice residue mulch alone except for pretilachlor (0.3 kg ai ha^-1^) which has a similar effect on weed emergence at 2 t ha^-1^ residue cover. In addition, the combined effects of pre-emergence herbicides with 2 t ha^-1^ residue were more detrimental to weed biomass accumulation than the 2 t ha^-1^ residue alone. Hence, the application of pre-emergence herbicides in combination with 6 t ha^-1^ residue, although effective, had a similar suppressive effect on *I*. *rugosum* emergence and biomass (root and shoot) accumulation.

However, pre-emergence herbicides can be intercepted by crop residue mulch present on the soil surface [38, 5]. Residue cover can be a physical barrier that separates the herbicides from the soil surface, with soil surface being the spot of herbicide incorporation, thus, may affect the herbicide’s inhibitory action on weed emergence. Therefore, herbicide efficacy is expected to be lower in these conditions than in soils with no residue cover [51]. Hence, evaluating the efficacy of some pre-emergence herbicides when applied with different amounts of residue cover could generate valuable information or provide insight on managing weeds, particularly *I*. *rugosum*.

### Response of *I*. *rugosum* seedlings to flooding depth and flood timing

**Fig 7:**
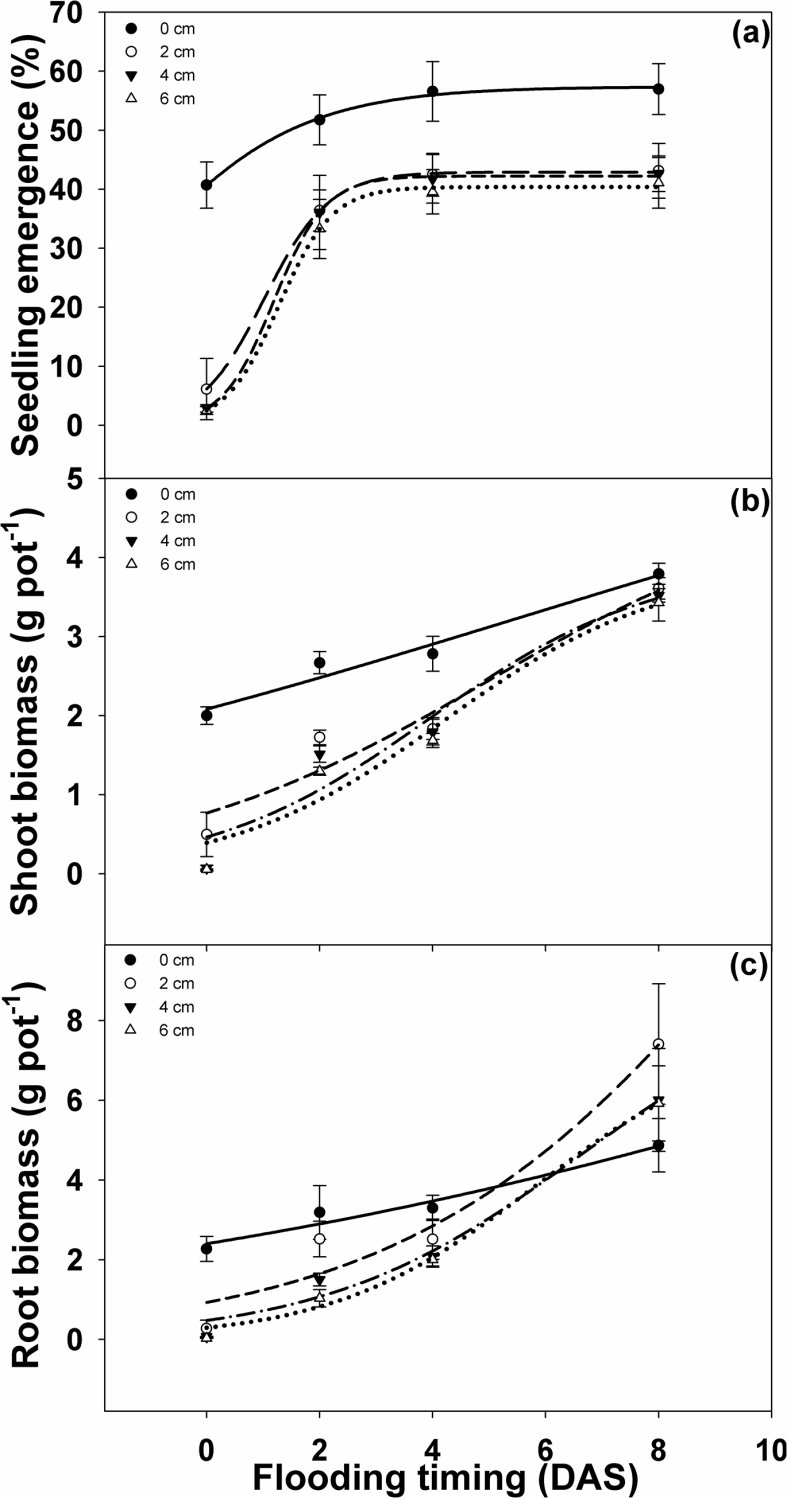
Effect of flood depth (cm) and time of flooding (d after sowing, DAS) on (a) *I*. *rugosum* seedling emergence (%), modeled with the use of equation *E* = *E*_*max*_/(1 + e ^[- (*x - T50*)/*Erate*]^; (b) *I*. *rugosum* shoot biomass (g pot^-1^); and (c) root biomass (g pot^-1^) under 21 d of continuous flooded condition, modeled with the use of equation *B* = *B*_*max*_/(1 + e ^[- (*x - T50*)/*Brate*]^. Estimated parameters are given in Table 4. Vertical bars represent standard error of the mean.

Flooding on the day of sowing (0 DAS) at depths of 2, 4, and 6 cm for 21 d reduced seedling emergence by 88, 94, and 95%, respectively. However, delay in flooding with respect to date of sowing resulted in less control on weed emergence. At the 2, 4, and 6 cm flood depths, weed emergence was reduced by 29, 35, and 36% when flooding at 2 DAS, by 24, 26, and 30% during flooding at 4 DAS, and by 24, 25, and 27% during flooding at 8 DAS, respectively (Fig 7A, Table 4).

**Table 4:** Effect of flood depth on seedling emergence shoot and root biomass of *I*. *rugosum* at various flood timing d after sowing. Parameter estimates [*E*_*max*_, maximum parameter (%); *T*_*50*_, flood time to reach 50% of maximum parameter (flood timing DAS); and *E*_*rate*_, slope] of a three-parameter sigmoid model {*E* = *E*_*max*_/(1 + e ^[- (*x - T50*)/*Erate*]^} fitted to seedling emergence data and biomass data in Fig 7, for biomass *E* replaces with *B*. Values in parentheses represent standard error of the mean.

Flooding depth (cm)	*E*_*max*_	*E*_*rate*_	*T*_*50*_	*R* ^*2*^
	**Seedling emergence coefficients**			
0	57.37 (0.74)	1.43 (0.21)	1.27 (0.20)	0.99
2	42.91 (0.24)	0.57 (0.01)	1.02 (0.03)	0.99
4	42.24 (0.40)	0.46 (0.03)	1.19 (0.05)	0.99
6	40.39 (0.70)	0.47 (0.05)	1.28 (0.09)	0.99
	**Shoot biomass coefficients**			
0	6.53 (9.89)	7.45 (9.86)	5.64 (23.25)	0.97
2	4.89 (4.37)	2.97 (2.98)	4.98 (6.48)	0.94
4	3.93 (1.66)	1.96 (1.72)	3.94 (2.54)	0.93
6	3.88 (1.49)	1.92 (1.47)	4.20 (2.28)	0.95
	**Root biomass coefficients**			
0	16.25 (95.69)	8.92 (15.61)	15.62 (87.85)	0.96
2	23.01 (149.84)	3.31 (5.05)	10.47 (35.37)	0.95
4	9.30 (10.19)	2.28 (1.81)	6.64 (5.87)	0.98
6	7.65 (2.83)	1.80 (0.78)	5.79 (1.92)	0.99

The individual main effects of flooding time and flood depth on weed root biomass were significant but the interaction effect on root biomass was not significant. Deeper and early flooding (0 DAS) reduced more weed root biomass than flooding 2 DAS or later. Flooding at 8 DAS increased root biomass by 89% compared to flooding on the day of sowing (Fig 7C, Table 4). On the other hand, flooding at 2 cm did not affect weed root biomass (only 4% reduction) compared to the non-flooded condition. Flooding at depths of 4 and 6 cm, however, reduced root biomass by 28 and 32%, respectively, compared to non-flooded conditions.

Time of flooding in combination with flood depth significantly affected weed shoot biomass (Fig 7B, Table 4). Flooding at 2–4 cm depth and on the day of sowing reduced weed shoot biomass more than in the non-flooded conditions. Flooding the seeds on the day of sowing reduced weed shoot biomass by 80, 96 and 98% when flooding at depths of 2, 4, and 6 cm, respectively. Flooding at depths of 2 and 4 cm at 2 DAS showed a similar effect on reduced weed shoot biomass (35 and 43%, respectively), while flooding at 6 cm reduced shoot biomass by 51%. whileFlooding at 8 DAS did not affect weed shoot biomass accumulation.

**Fig 8:**
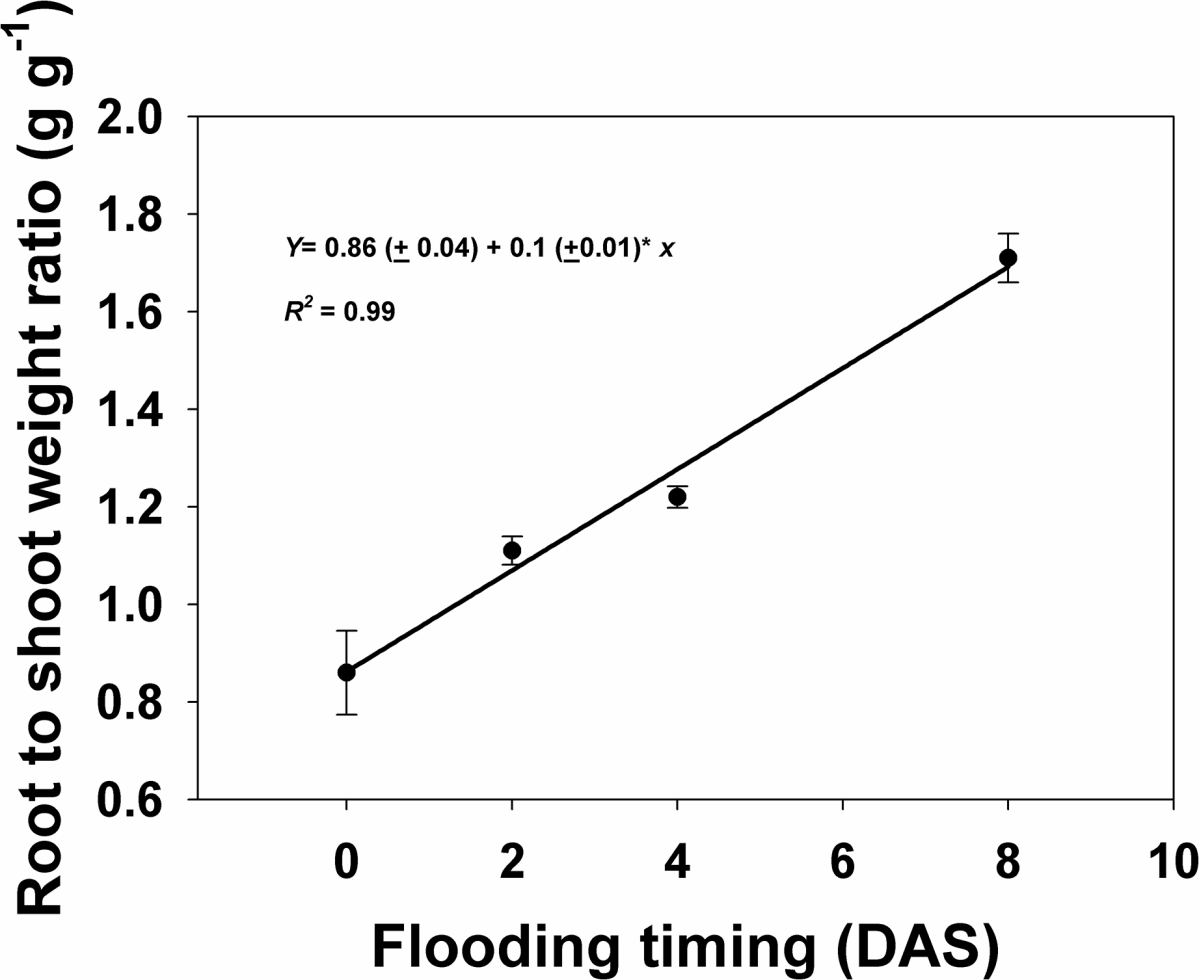
Interaction effect of flood depth (cm) and time of flooding (in days after sowing) on weed root-shoot weight ratio of *I*. *rugosum* after 21 d of continuous flooding, modeled using the equation *y = y0+ax*. Vertical bars represent standard error of the mean.

Treatment effect on *I*. *rugosum* root-shoot weight ratio was only significant at the time of flooding treatment. Early flooding significantly reduced the root–shoot biomass ratio (Fig 8). Flooding the seeds on the day of sowing and at 2 DAS reduced the weed root–shoot biomass ratio by 41 and 28%, respectively, compared to flooding at 8 DAS. On the other hand, flooding at 4 DAS did not reduce the weed root–shoot biomass ratio.

Although flooding significantly reduced weed emergence compared to non-flooded conditions, early flooding appeared to be the most effective strategy in suppressing the emergence of *I*. *rugosum*. Root biomass was not affected by the interaction between flood depth and time of flooding. Instead, it was affected by flooding depths of 4 and 6 cm which lowered it. Flooding on the day of sowing proved to be the most effective treatment in reducing root growth and further delay in flooding would allow more root growth. Shoot biomass was severely affected by flood depth in combination with the time of flooding. Although the effects of flood depths on shoot biomass were similar, flooding within 4 DAS may significantly suppress shoot biomass production but flooding at 8 DAS will not. Weed root–shoot biomass ratio was lowest when flooding was introduced at 0 and 2 DAS, and flooding at 4 DAS or onward did not reduce the root–shoot biomass ratio.

*I*. *rugosum* weed emergence and shoot biomass were effectively controlled by the combined effect of the timing and depth of flooding. Weed root biomass and root–shoot ratio were affected by either depth or timing of flooding. Hence, early flooding is the most effective strategy in controlling *I*. *rugosum* emergence and growth even at shallow flood depths. Flooding should be imposed to control weeds [4], although not all farms have readily available and accessible water in time for an effective flooding strategy. Thus, water availability is the limiting factor when using flooding as a strategy in controlling weeds.

The rice crop is more tolerant to flooding than many weed species, making flooding a viable management strategy against weeds [5]. The timing, duration, and depth of flooding determine the specific nature of weed suppression by flooding [52]. A 72% reduction in emergence is reported when *L*. *chinensis* was subjected to 7 d of 2 cm continuous flooding compared to the 26% decline in emergence when flooded for 2 d out of 7 d at the same flooding depth [32]. The same study showed that biomass production was significantly reduced by 73 and 99%, respectively, when *L*. *chinensis* was flooded for 2 d and 7 d at 2 cm water depth. In another study, flooding at 1 day after application of propanil reduced the germination of *I*. *rugosum* seeds under wetland conditions [14]. The application of the herbicide pretilachlor in combination with flooding has been shown to effectively suppress the seedling emergence and growth of *Cyperus difformis* L., *Ludwigia hyssopifolia* (G. Don) Exell, and *Echinochloa colona* [53].

### Seedling response of *I*. *rugosum* to different rates of pretilachlor and flood depth

**Fig 9:**
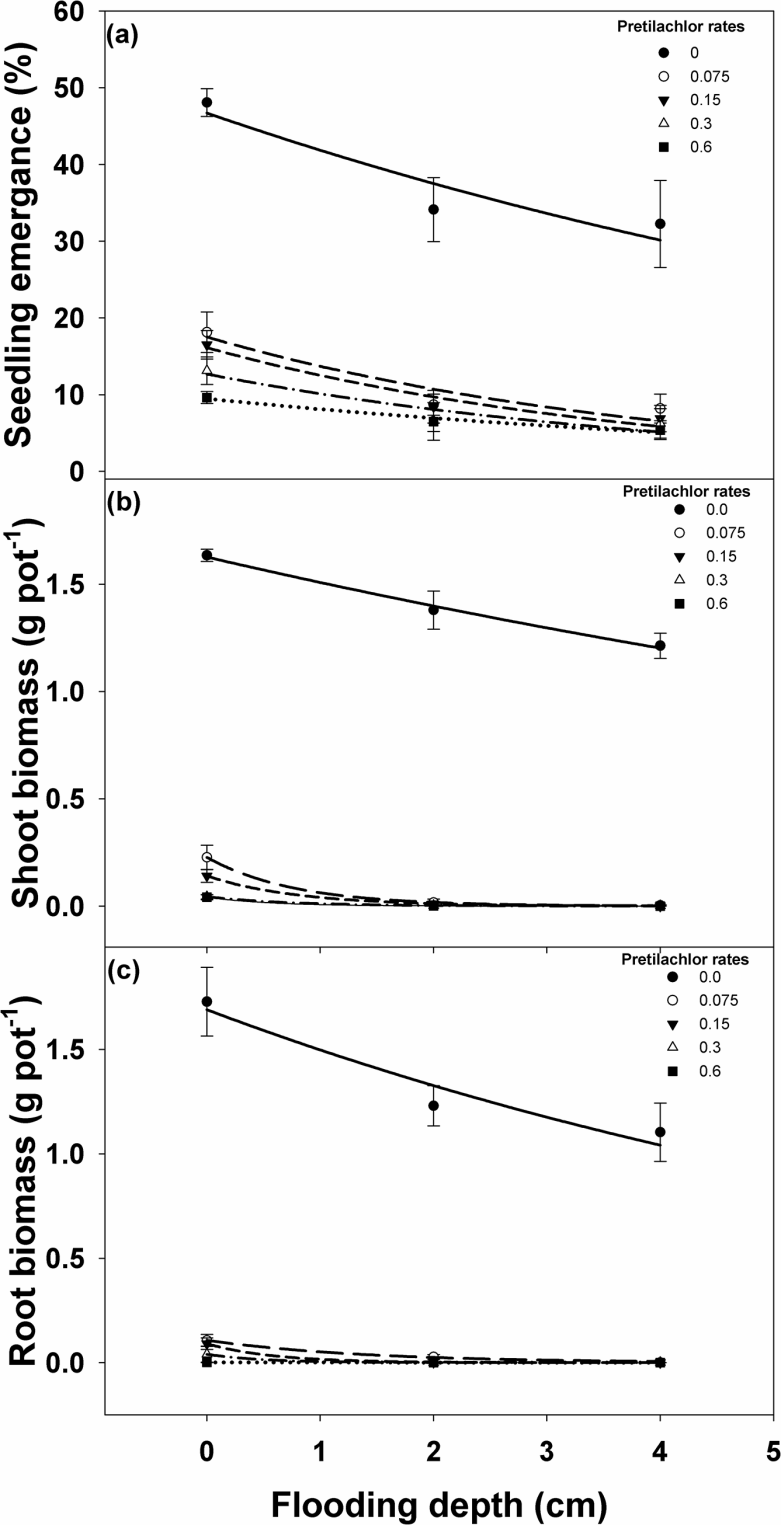
Effect of flood depth and pretilachlor rates (kg g.a.i.) on (a) *I*. *rugosum* seedling emergence, modeled with the use of equation *E* = *E*_*max*_ * *e* ^(-Erate . x)^; (b) *I*. *rugosum* seedling shoot biomass; and (c) root biomass after 21 days of continuous flooding conditions, modeled with the use of equation *B* = *B*_*max*_ * *e* ^(-Brate . x)^. Estimated parameters are given in Table 5. Vertical bars represent standard error of the mean.

Weed emergence and biomass production were affected by flooding in combination with pretilachlor application. Differences in weed emergence were significant among pretilachlor application rates, flood depths, and pretilachlor application rate by flood depth interaction (Fig 9, Table 5). Flooding at depths of 2 and 4 cm without pretilachlor application reduced weed emergence to 34 and 29%, respectively.

**Table 5:** Effect of different pretilachlor herbicide rates (kg a.i. ha^-1^) on the seedling emergence, of *I*. *rugosum*. Parameter estimates [*E*_*max*_, maximum emergence (%) and *E*_*rate*_, slope] of a two-parameter exponential model (*E* = *E*_*max*_ * e ^(-*Erate . x*)^) fitted to seedling emergence, root, and shoot biomass data in Fig 9. Values in parentheses represent standard error of the mean.

Pretilachlor rate (kg a.i. ha^-1^)	*E*_*max*_	*E*_*rate*_	*R* ^*2*^
	**Seedling emergence coefficients**		
0.0	46.70(3.99)	0.11(0.04)	0.88
0.075	17.51(2.60)	0.25(0.09)	0.89
0.15	16.12(1.64)	0.25(0.07)	0.89
0.3	12.67(1.82)	0.23(0.09)	0.88
0.6	9.47(0.53)	0.16(0.03)	0.96
	**Shoot biomass coefficients**		
0	1.63(0.02	0.08(0.01)	0.99
0.075	0.23(0.00)	1.30(0.05)	0.99
0.15	0.04(0.00)	1.39(0.15)	0.99
0.3	0.04(0.00)	1.76(0.15)	0.99
0.6	Model did not fit		
	**Root biomass coefficients**		
0	1.69(0.12)	0.12(0.03)	0.93
0.075	0.11(0.01)	0.74(0.10)	0.99
0.15	0.09(0.00)	1.77(0.08)	0.99
0.3	0.04(0.00)	2.09(0.08)	0.99
0.6	0.00(0.00)	0.63(0.06)	0.99

Pretilachlor application significantly reduced weed emergence compared to the control (no flood and no pretilachlor application). In the non-flooded treatment, the application of pretilachlor at 0.075 and 0.150 kg ai ha^-1^ reduced weed emergence by 61 and 65%, respectively. The application of pretilachlor at 0.225 and 0.3 kg ai ha^-1^ reduced weed emergence by 72 and 79%, respectively. When the weed seeds were flooded at a depth of 2 cm, pretilachlor application significantly reduced weed emergence but the reduction did not significantly differ within application rates. At a 2 cm flood depth, application of pretilachlor at 0.3 kg ai ha^-1^ had the highest weed emergence reduction (80%) while the application of 0.075 kg ai ha^-1^ provided the least emergence reduction (74%) relative to the control. When weed seeds were flooded at a depth of 4 cm after pretilachlor application, weed emergence was reduced in all pretilachlor application rates compared to the control. However, differences in emergence reduction between pretilachlor rates did not differ in the 4 cm flooding depth. Compared to the control, the most reduction on *I*. *rugosum* emergence was 83% at 0.6 kg ai ha^-1^ pretilachlor application and the least was 75% at 0.075 kg ai ha^-1^ application rate when flooded at 4 cm depth after pretilachlor application.

Root biomass differed with pretilachlor rate, flood depths, and pretilachlor rate by flood depth interaction (Fig 9C, Table 5). Flooding alone at depths of 2 and 4 cm reduced the weed root biomass by 28 and 36%, respectively, compared to the control. On the other hand, the application of pretilachlor alone at increasing rates also reduced the weed root biomass by 93–99%, with the 0.075 kg ai ha^-1^ rate having the least reduction effect and the 0.3 kg ai ha^-1^ rate having the most root biomass reduction effect compared to the control. The application of increasing rates of pretilachlor a day after sowing followed by flooding further reduced the weed root biomass by 97–99% at 2 cm flood depth and by 99% at 4 cm flood depth in all pretilachlor rates.

Shoot biomass also differed with pretilachlor rates, flood depths, and pretilachlor rate by flood depth interactions (Fig 9B, Table 5). Flooding at depths of 2 and 4 cm alone significantly reduced weed shoot biomass by 15 and 25%, respectively. Under the non-flooded condition, the application of pretilachlor rates significantly reduced weed shoot biomass by as much as 91% at 0.150 kg ai ha^-1^, while increasing the pretilachlor rate to 0.3 kg ai ha^-1^ reduced weed shoot biomass by 97% relative to the control. Flooding at depths of 2 and 4 cm after application of pretilachlor greatly reduced weed shoot biomass by 98–99%, although the effect of increasing pretilachlor rates from 0.075 to 0.3 kg ai ha^-1^ did not differ at the 2 cm flooding depths.

Flooding at 2 and 4 cm depths had a significant effect on emergence and biomass reduction in *I*. *rugosum*. Increasing the pretilachlor rate from 0.075 to 0.3 kg ai ha^-1^ had similar reduction effects on weed emergence and biomass. In this study, a lower weed emergence was observed at the 0.3 kg ai ha^-1^ pretilachlor application rate. This was due to a slight change in observation in that most of the seeds under this treatment produced plumule but this dried up and died on the last observation day. While few had their plumule remain green, these were stunted and did not develop into fully normal seedlings. Thus, seedling emergence was considered only in seedlings with plumules that remained green.

Flooding in combination with pretilachlor application is more effective in suppressing weed emergence and biomass production than sole flooding or pretilachlor application alone. Other weed species differ in their response to pretilachlor. The emergence of *Echinochloa* species is not affected by pretilachlor application, but its biomass production is highly correlated with low amylase activity because of pretilachlor application [54]. Flooding reduces oxygen levels and can alter the quality and quantity of light in murky waters. Thus, flooding can inhibit *I*. *rugosum* emergence and growth, and will become more effective when combined with pre-emergence herbicide application, hence, controlling *I*. *rugosum* more effectively.

The effect of intermittent flooding and pretilachlor rates on emergence and early growth of three common weeds of lowland rice was assessed in a screenhouse study. Junglerice (*Echinochloa colona*), smallflower umbrella sedge (*Cyperus difformis*), and ludwigia (*Ludwigia hyssopifolia*) were treated with pretilachlor rates ranging from 0.075 to 0.3 kg a.i. ha^-1^ and subjected to alternate flooding (0, 2, 4, and 6 cm water depth). Intermittent flooding to 6 cm decreased the emergence of all three weed species. Smallflower umbrella sedge growth was not affected by flooding alone, but was completely suppressed by pretilachlor application. The growth of junglerice was reduced more by pretilachlor when application was followed by flooding [53].

Similar findings were reported on other weed species wherein increasing rates of pretilachlor from 0–0.9 kg a.i ha^-1^ reduced the seedling emergence and seedling biomass of *E*. *colona* [55]. In an earlier study on three pre-emergence herbicides (oxadiazon, butachlor, and acetachlor), oxadiazon and butachlor reduced weed emergence by 90.2 and 84.2% relative to the untreated plot. The performance of acetachlor was poor compared to oxadiazon and butachlor [56]. In another study, three herbicides (oryzalin, trifluralin, and pendimethalin) were effective in reducing the plant emergence and biomass production of rigid ryegrass, with oryzalin as the least effective as compared to the other herbicides [57].

## Conclusion

Light is required for the germination of *I*. *rugosum* seeds. Warmer day/night temperatures (35/25°C) can promote or accelerate seed germination, while cooler temperatures (25/15°C) may inhibit it. Low salinity and mild drought (-0.2 MPa) environmental stresses can easily hamper the germination of *I*. *rugosum* seeds.

Cultural management practices such as seed burial through shallow tillage and the use of rice residue as mulch can significantly suppress seedling emergence and growth, particularly when residue mulching is done in combination with pre-emergence herbicide application. Flooding at shallower depths (2 cm) can also suppress seedling emergence and growth, while early flooding (0 DAS) has a more pronounced suppression effect on *I*. *rugosum*, particularly when flooding is done in combination with pretilachlor application.

Although *I*. *rugosum* is a highly competitive weed of rice in direct-seeded conditions, this study showed that such cultural management practices can contribute to the effective control of *I*. *rugosum*. These practices under field conditions, however, may require different strategy and weed management combinations to maximize weed control efficacy. Implementing these cultural management practices needs a considerable amount of labor, materials, and other resources like water for flooding, which should all be taken into account at the field level to achieve attainable but effective control strategies. Further studies on the effective control of the weed under field conditions should be pursued not only to validate the results of the present study but also to provide a complete understanding on the nature and effective management of *I*. *rugosum*.
